# Reinforcement learning-based dynamic field exploration and reconstruction using multi-robot systems for environmental monitoring

**DOI:** 10.3389/frobt.2025.1492526

**Published:** 2025-03-25

**Authors:** Thinh Lu, Divyam Sobti, Deepak Talwar, Wencen Wu

**Affiliations:** Computer Engineering Department, San Jose State University, San Jose, CA, United States

**Keywords:** multi-robot systems, mobile sensor networks, reinforcement learning, dynamic field reconstruction, source seeking, environmental monitoring

## Abstract

In the realm of real-time environmental monitoring and hazard detection, multi-robot systems present a promising solution for exploring and mapping dynamic fields, particularly in scenarios where human intervention poses safety risks. This research introduces a strategy for path planning and control of a group of mobile sensing robots to efficiently explore and reconstruct a dynamic field consisting of multiple non-overlapping diffusion sources. Our approach integrates a reinforcement learning-based path planning algorithm to guide the multi-robot formation in identifying diffusion sources, with a clustering-based method for destination selection once a new source is detected, to enhance coverage and accelerate exploration in unknown environments. Simulation results and real-world laboratory experiments demonstrate the effectiveness of our approach in exploring and reconstructing dynamic fields. This study advances the field of multi-robot systems in environmental monitoring and has practical implications for rescue missions and field explorations.

## 1 Introduction

Environmental monitoring, including the identification and tracing of areas impacted by environmental hazards, is paramount for safeguarding human life and property. Early warning systems allow for swift responses to potential threats. Effective environmental monitoring relies on a deep understanding of key processes like wildfire propagation and pollutant dispersion. These phenomena often involve spatial and temporal changes, making them suitable for modeling using partial differential equations (PDEs). For instance, the advection-diffusion equation can be used to simulate the movement of smoke plumes from wildfires, providing crucial insights for predicting their evolution over time (Khaled et al., 2004; Reisch et al., 2024). This information is essential for effective environmental hazard monitoring and mitigation.

For environmental monitoring tasks, multi-robot systems offer significant advantages over single-robot setups by enabling faster coverage of larger areas and providing redundancy against individual failures. These systems excel in complex missions across diverse environments, including search and rescue (Niroui et al., 2019; Shuvo et al., 2023; Cao et al., 2024), underwater surveillance (Martins et al., 2018; Luvisutto et al., 2022), and space exploration (Gautam et al., 2019; Bi et al., 2024; Long and Zhang, 2024). These works place additional emphasis on developing robust coordination strategies and efficient path-planning algorithms. Coordination may be centralized, with a leader directing actions, or decentralized, with robots making their own decisions based on local observations. Reinforcement learning has advanced these strategies, with actor–critic models enhancing control stability of the whole unit under dynamic disturbances (Hu et al., 2023) and graph-based methods enabling scalable, distributed decision-making across large robot teams (Chen et al., 2024). Depending on the mission, formation control may also play an essential role, where the robot system can be operated in organized patterns for high-quality data collection, or independently for greater flexibility. Beyond coordination, reinforcement learning-based approaches have also been increasingly adopted for path planning, further enhancing adaptability and performance of multi-robot systems in unknown environments (Zhu et al., 2023). These approaches require careful design of both the simulation environment and reward functions, which should closely model real-world conditions, to ensure effective learning and reliable performance in deployment. For applications in environmental monitoring, multi-robot systems can be equipped with specialized sensors to enable real-time data collection and reconstruction of environmental processes (Kinaneva et al., 2019; Dunbabin and Marques, 2012; Queralta et al., 2020; Rossi and Brunelli, 2016).

To reconstruct dynamic processes through limited measurements from multi-robot systems, it is necessary to identify unknown parameters in the PDEs that describe these processes, such as the diffusion coefficient in a diffusion equation. A common approach is to deploy static sensor networks (Mourikis and Roumeliotis, 2006; Burgard et al., 2005). Although effective, this approach is both costly and impractical for large-scale regions due to the need for extensive sensor installations. Mobile sensor networks, with collaborative mobile sensing robots, present a more practical alternative, offering great flexibility and broad coverage while using fewer sensors. In mobile sensor networks, parameter identification can be performed in two primary ways: offline and online (Zhang et al., 2023). Offline parameter identification requires mobile sensor networks to explore the entire spatial domain before any parameter estimation begins (Ucinski, 2005; Ucinski and Chen, 2005; Tricaud and Chen, 2010). This approach often uses techniques like least squares optimization to minimize the error between the observed and estimated states, typically requiring complex computations to solve PDEs. While this approach generally yields more accurate results, it is time-consuming, as full data collection must be completed before any estimation can take place. Due to the limitations of offline methods, increasing attention has shifted toward online parameter identification approaches (Wu et al., 2020; Zhang et al., 2023; Christopoulos and Roumeliotis, 2005). Online identification continuously updates parameter estimates as mobile sensors collect data in real time. While this approach may not provide the most accurate solution to PDEs compared to offline methods, it is far more efficient for time-sensitive applications like environmental hazard management (Zhang et al., 2023).

A key challenge of online parameter identification is determining an information-rich trajectory for the mobile sensing network, as this directly impacts the speed and accuracy of field reconstruction. However, since online methods operate in real-time, predicting the optimal path in advance is challenging, making efficient trajectory planning a complex problem. As a result, recent works in this field often provide additional strategies for effective trajectory planning and navigation for mobile sensor networks. In (You et al., 2016; Zhang et al., 2023), the authors employ a cooperative Kalman filter (CKF) combined with recursive least squares (RLS) to identify advection-diffusion field parameters in real-time using live sensor readings from a formation of mobile robots. To ensure that the robot formation follows information-rich trajectories, several studies, including (You and Wu, 2018; You et al., 2022), have integrated robot dynamics into the field dynamics and focused on minimizing mapping errors. However, these approaches may converge to local optima and may not adequately address the complexity of field reconstruction in environments involving multiple diffusion fields with varying characteristics. To address this issue, The author in (Talwar, 2020) proposes an exploration strategy that samples nearby candidate destinations based on custom weights calculated from cosine similarity to the centroid of unvisited regions and distance from explored diffusion fields. However, this approach may result in inefficient backtracking and revisits, which are undesirable in time-critical missions.

To tackle the problem of exploring complex dynamic fields, this research introduces a strategy for path planning and control of mobile sensing robots designed to effectively explore and reconstruct a dynamic field consisting of multiple non-overlapping diffusion fields while offering a good balance between speed and accuracy. In our proposed algorithm, the robot formation alternates between two primary operational modes: Field Exploration and Source Mapping. In Source Mapping mode, the formation makes use of reinforcement learning (RL), specifically, proximal policy optimization (PPO) to direct the robot formation to the center of a newly discovered diffusion field, while attempting to estimate its diffusion and advection coefficients through the CKF and RLS developed in (You et al., 2022). When dealing with the challenging problem that multiple sources exist in the field and the path planned in Source Mapping mode only leads to one source (local maximum) in the field, we develop a novel K-means clustering algorithm in the Field Exploration mode, to allow the robot formation advances toward unexplored regions to identify traces of potential new diffusion fields. The K-means clustering algorithm is used to partition the unexplored regions and facilitate faster scanning of the whole map. We validate our proposed strategy through both computer simulations and controlled laboratory experiments. In these scenarios, the robot formation is randomly placed within a spatially and temporally varying field, and we compare the field reconstruction errors to baseline strategies that employ random or lawn-mowing trajectories. Our research demonstrates the potential of multi-robot formations for accurate field reconstruction in complex environments characterized by multiple spatial-temporal diffusion fields.

To summarize, the main contributions of the paper are twofold: (1) it introduces a novel two-mode strategy for path planning and control of mobile sensing robots in dynamic environments, specifically for exploring and reconstructing fields with multiple non-overlapping diffusion sources. The strategy integrates RL-based path planning with a CKF and RLS for estimating unknown parameters of the field. A key innovation is the use of K-means clustering algorithm to facilitate efficient exploration of unexplored regions, ensuring a balance between speed and accuracy. (2) Through both simulations and controlled experiments, the research demonstrates the effectiveness of the proposed strategy in improving field reconstruction accuracy using only a limited number of mobile sensing robots.

The remainder of this paper is structured as follows. In Section 2, we formally define the problem. We present some preliminary information in Section 3. The proposed algorithm is presented in Section 4, followed by a detailed analysis of the simulation and experimental results in Section 5 and Section 6 respectively. Finally, Section 7 summarizes our findings and outlines future research directions.

## 2 Problem formulation

In this section, we formulate the problem of reconstructing an unknown spatial-temporal varying field represented by a linear combination of several advection-diffusion equations, using a team of mobile sensing robots.

### 2.1 Spatial-temporal varying fields

Various processes that exhibit spatial and temporal variations, such as the dispersion of pollutants in the atmosphere or water bodies, are often represented by two-dimensional (2D) PDEs over a domain R . A typical example is the 2D advection-diffusion equation, which models the transfer of substances via advection (the movement of substances through a fluid) and diffusion (the spreading of substances from areas of higher to lower concentration). This can be mathematically expressed as:

$$\frac{\partial z}{\partial t}\left(r,t\right)=\theta \nabla^{2}z\left(r,t\right)+v^{T}\nabla z\left(r,t\right),\;r\in R,$$ (1)

where z(r,t) denotes the concentration function of the field at position r at time instance t , ∇ and $\nabla^{2}$ are the gradient and Laplacian operators, respectively. The coefficient θ>0 represents the rate of diffusion, and v is the two-dimensional advection coefficient, representing the speed at which a quantity such as heat, concentration, or pollutant is transported by the bulk movement of a fluid. Both θ and v are considered constant but possibly unknown over a fixed interval.

In this work, we consider the field as a linear superposition of multiple non-overlapping advection diffusion phenomena, each governing a spatial-temporal region $R_{i},\;i=1,\ldots ,M$ where M is the number of the advection-diffusion processes. The concentration *z_i(r,t)_* in each region satisfies the advection-diffusion equation: $\frac{\partial z_{i}}{\partial t}\left(r,t\right)=\theta \nabla^{2}z_{i}\left(r,t\right)+v^{T}\nabla z_{i}\left(r,t\right),\;r\in R_{i}$ . The global concentration field is then represented as

$$z\left(r,t\right)=\underset{i}{\sum }\chi_{i}\left(r\right)z_{i}\left(r,t\right),\;r\in \Omega ,$$ (2)

where *χ_i_* (*r*) is an indicator function defined as $\chi_{i}\left(r\right)=1$ if $r\in R_{i}$ and 0 otherwise, and $\Omega =⋃R_{i},\;i=1,\ldots ,M$ . Moreover, in various real-world environmental monitoring scenarios, the domain Ω is significantly larger than the dimensions of the operational robots, enabling the approximation of the boundary as essentially flat. Under these conditions, we apply the initial and Dirichlet boundary conditions as shown in Equation 3 at the boundary ∂Ω (Demetriou et al., 2013):

$$\begin{array}{cc} z\left(r,0\right) & =z_{0}\left(r\right),\\ z\left(r,t\right) & =0,\;r\in \partial \Omega . \end{array}$$ (3)

### 2.2 Mobile sensing robots

In this work, we consider a group of N mobile sensing robots moving in a coordinated formation in the field Ω represented in Equation 2. The algorithm proposed in this work commands the formation to travel on planned paths to efficiently reconstruct the unknown field. We make the following assumption regarding these mobile sensing robots.

Assumption 2.1 *Each sensing robot is equipped with sensors to localize itself and to measure the field concentration value at its current location at each discrete time step* k .

The measurement of the i th sensing robot at time step k is modeled as follows:

$$p\left(r_{i}^{k},k\right)=z\left(r_{i}^{k},k\right)+n_{i},\;i=1,\ldots N,$$ (4)

where $r_{i}^{k}$ represents the location of the i th robot at the discrete time step k and $n_{i}$ is assumed to be i.i.d Gaussian noise. Additionally, using the locations of all the robots in the formation at time step k , we can determine the location of the formation center $r_{c}^{k}$ at time step k as $r_{c}^{k}=\frac{1}{N}\sum_{i}^{N}r_{i}^{k}$ .

**FIGURE 1:**
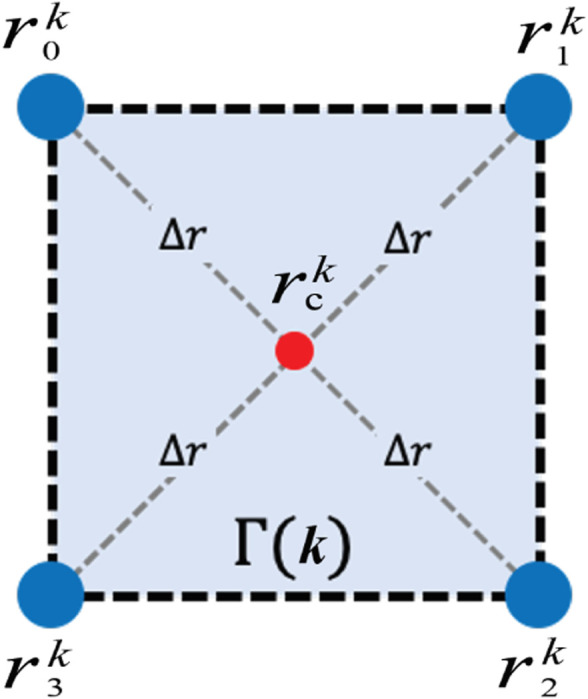
A symmetric formation composed of four mobile robots $r_{i}^{k},\;i=0,1,2,3$ shown in blue. The formation center $r_{c}^{k}$ is shown in red. The distance between each robot and the formation center is Δr . The shaded region is the time-varying view-scope Γ(k) .

When the robots move in a desired formation, it covers a time-varying view-scope Γ(k) , which is the area of the field domain Ω that lies inside the polygon formed by sensing robot locations. As shown in Figure 1, the shaded region illustrates the time-varying view-scope Γ(k) at discrete time step k , the blue circles represent the four mobile robots, and the red circle represents the formation center. At any given time, the mobile sensing robots can measure and exchange concentration values at their specific locations as shown in Equation 4 and the field values $z\left(r^{k},k\right),\;r^{k}\in \Gamma \left(k\right)$ can be estimated by interpolating the measured values from the robots. Consequently, it is reasonable to assume that the estimated field values, $z\left(r^{k},k\right),r^{k}\in \Gamma \left(k\right)$ , are available to us at all times.

In this work, to facilitate the implementation of the PPO algorithm for source mapping in Section 4.1, we discretize the global field Ω to a E×F grid, where each grid cell represents a single location $r^{k}$ in the map and associates with a concentration value $z\left(r^{k},k\right)$ . The following assumption holds for the formation center.

Assumption 2.2 *Robots travel in a coordinate formation and the formation center moves along the eight possible directions “up”, “down”, “left”, “right”, “up-left”, “up-right”, “down-left”, “down-right” in the discretized domain.*

With the robots moving in a formation, a CKF developed in (You et al., 2016; Wu et al., 2020) is employed to output estimates of concentration $z\left(r_{c}^{k},k\right)$ and gradients $\nabla z\left(r_{c}^{k},k\right)$ at the formation center $r_{c}$ at time step k . These estimated values will play a major role in the developed algorithm in Section 4. Furthermore, we apply the parameter identification algorithms developed in (Wu et al., 2020) to estimate the unknown diffusion coefficient θ in real-time using the output from the CKF and the RLS algorithm. Thus, in the following discussions, we consider θ as a known value for field reconstruction.

Remark 2.1 *Multi-robot formation control is a well-studied topic and researchers have developed numerous formation control algorithms* (Zhang and Leonard, 2010; Ren and Beard, 2008; Wu and Zhang, 2012)*. In this work, we employ the formation control strategy developed in* (Zhang and Leonard, 2010) *and applied in* (Wu et al., 2020)*. The strategy uses the Jacobi transform to decouple the formation control from the motion control of the multi-robot formation, which enables us to only plan the path and design the controller for the formation center* $r_{c}$ *. The individual robot controllers are then derived using the formation controller.*

### 2.3 The multi-robot source seeking and field reconstruction problem

In real-world scenarios, the task of mapping complex dynamic fields for cases like gas-leaking and wildfires is important and is often time-critical. It is essential for the robot formation to explore and detect diffusion sources in unknown areas and generate a map as quickly as possible. With the field defined in Section 2.1 and the multi-robot formation defined in Section 2.2, the goal of this study is to design a path for the multi-robot formation so that the formation can identify the multiple non-overlapping diffusion sources in the dynamic field and reconstruct the field in real-time with the limited concentration measurements collected by the multi-robot formation along its trajectory. To achieve the goal, we will introduce a two-mode strategy in Section 4, which consists of a Source Mapping mode and a Field Exploration mode. In the Source Mapping mode, we employ the RL-based algorithm and train a PPO model to guide the multi-robot formation toward a diffusion source in the field and reach a stationary state, where the formation arrives at the source and moves with the field at the same speed as the advection flow. In the Field Exploration mode, we develop a K-means clustering-based exploration strategy to enable efficient exploration of unknown areas.

## 3 Preliminaries

### 3.1 Proximal policy optimization

PPO (Schulman et al., 2017) is a significant development in reinforcement learning, introduced as a more efficient and simpler alternative to Trust Region Policy Optimization (TRPO) (Schulman et al., 2015). PPO is based on the policy gradient approach, a class of algorithms that optimize policies by directly computing gradients of expected rewards for policy parameters. This approach allows the learning agent to improve its policy iteratively by following the gradient of expected rewards. PPO enhances this process by addressing the complexities of earlier methods while retaining their benefits, particularly in maintaining stable and reliable policy updates.

PPO operates as an on-policy method. Unlike traditional policy gradient methods that apply a single update after each interaction with the environment, PPO refines the policy by using multiple updates on the same batch of data. The core of PPO is its surrogate objective function, designed to prevent large, potentially destructive policy updates. This is achieved through a probability ratio $r_{k}$ between the new and old policies, which is clipped to keep updates within a safe range. The surrogate objective function $L^{\mathrm{CLIP}}\left(\theta \right)$ is expressed as:

$$L^{CLIP}\left(\theta \right)={\hat{E}}_{k}\left[\min\left(r_{k}\left(\theta \right){\hat{A}}_{k},\mathrm{clip}\left(r_{k}\left(\theta \right),1-\varepsilon ,1+\varepsilon \right){\hat{A}}_{k}\right)\right].$$ (5)

In Equation 5, ${\hat{E}}_{k}$ denotes the expectation over timestep k , θ represents the policy parameters, ${\hat{A}}_{k}$ is an estimate of the advantage function at time step k , and ε is a small hyperparameter that controls the clipping range. It is important to note that while $r_{k}$ and θ follow the conventional notations used in literature, they differ from the notations in other sections of this paper, where r refers to the locations in the field and θ refers to the diffusion coefficient. The clipping mechanism ensures that if the probability ratio deviates outside the predefined range [1−ε,1+ε] , the function applies the clipped values to prevent excessively large updates, thereby maintaining the stability of the learning process. By constraining the probability ratio, PPO effectively controls the size of policy updates, balancing stability and performance. PPO is particularly well-suited for discrete action spaces, which makes it an ideal choice for our environment setup. The PPO algorithm is summarized in Algorithm 1.

Algorithm 1 PPO-Clip Algorithm.

**Input:** Initial policy parameters $\theta_{0}$ , initial value function parameters $\phi_{0}$

**for** k=0,1,2,…  **do**
  Generate a set of trajectories $D_{k}=\left\{\tau_{i}\right\}$ by running the policy $\pi_{k}=\pi \left(\theta_{k}\right)$ in the environment
  Calculate the rewards-to-go ${\hat{R}}_{k}$

  Estimate advantages ${\hat{A}}_{k}$ using a suitable method based on the current value function $V_{\phi_{k}}$

  Update the policy by maximizing the PPO-Clip objective
$$\theta_{k+1}=\arg\,\max_{\theta }\,\frac{1}{|D_{k}|T}\sum_{\tau \in D_{k}}\sum_{k=0}^{T}\min\left(r_{k}\,\left(\theta \right)\,{\hat{A}}_{k},\mathrm{clip}\left(r_{k}\,\left(\theta \right),1-\varepsilon ,1+\varepsilon \right)\,{\hat{A}}_{k}\right),$$
 where $r_{k}\left(\theta \right)=\frac{\pi_{\theta }\left(a_{k}|s_{k}\right)}{\pi_{\theta_{k}}\left(a_{k}|s_{k}\right)}$ .
  Update the value function by minimizing the mean-squared error:
$$\phi_{k+1}=\arg\;\underset{\phi }{\min}\frac{1}{|D_{k}|T}\underset{\tau \in D_{k}}{\sum }\overset{T}{\underset{k=0}{\sum }}{\left(V_{\phi }\left(s_{k}\right)-{\hat{R}}_{k}\right)}^{2}.$$

### 3.2 Cooperative Kalman Filter

Cooperative Kalman Filter (CKF) is a collaborative state estimation scheme first developed in (Zhang and Leonard, 2010), then used in later studies (Wu and Zhang, 2012; You et al., 2016; Wu et al., 2020; You et al., 2022), by combining live sensor data collected by the network of multiple mobile robots to collaboratively improve the accuracy of the state estimation process. In particular, when applied to the state estimation in dynamic fields, the authors incorporated the dynamics of the mobile robot formation and the diffusion equation into the formulation of the state equation of the CKF. This integration facilitates reliable and accurate state estimation, taking into account how changes in diffusion fields and the formation trajectory over time affect sensor data measurements. More specifically, the state vector X(k) at each time step k is defined as:

$$X\left(k\right)={\left[z\left(r_{c}^{k},k\right),\nabla z\left(r_{c}^{k},k\right),z\left(r_{c}^{k},k-1\right),\nabla z\left(r_{c}^{k},k-1\right)\right]}^{T},$$

where $z\left(r_{c}^{k},k\right)$ and $z\left(r_{c}^{k},k-1\right)$ denote the field concentration values at location $r_{c}^{k}$ at two consecutive time steps k and k−1 , respectively, and $\nabla z\left(r_{c}^{k},k\right)$ and $\nabla z\left(r_{c}^{k},k-1\right)$ denote the field gradient at location $r_{c}^{k}$ at two consecutive time steps k and k−1 , respectively. Given that the mobile robots maintain a symmetrical formation while traversing the environment, CKF estimates the state vector X(k) along the trajectory of the formation center. Note that since the field is spatial-temporal varying, the field concentration values and gradients are different at time steps k and k−1 even at the same location $r_{c}^{k}$ . This fact is critical in the construction of the CKF to provide reliable estimates of the state vector. The estimated state vector is subsequently employed to iteratively identify the diffusion coefficients of the field over time, based on the RLS algorithm. The estimated diffusion coefficients are vital for the task of identifying and reconstructing spatial diffusion fields in our paper. To save time and avoid excessive length in this paper, we will not provide the complete derivation of the CKF. For additional details, interested readers may refer to the original papers.

## 4 Methodology

In this section, we introduce the proposed path-planning algorithm for guiding the mobile sensing robot formation to quickly explore an open field while reliably mapping and reconstructing all detected diffusion sources along its trajectory. The algorithm aims to find a balance between speed and reliability for the dynamic field reconstruction. To achieve this, the solution alternates the robot formation between two operational modes: Map Exploration and Source Mapping. In Map Exploration, the robots systematically advance toward unexplored regions to detect new diffusion fields. Upon detecting a new diffusion field, the system transitions to Source Mapping, where the formation converges on the field’s center to achieve a stationary state, necessary for estimating advection coefficients.

**FIGURE 2:**
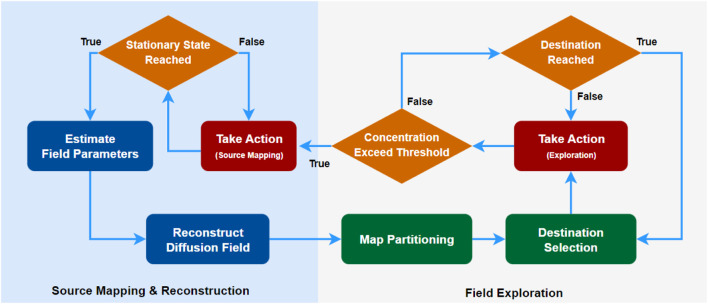
Flow chart showing the key components of our algorithm and two operation modes of the robot formation.

Throughout both modes, the robots continuously collect data, using the CKF for real-time concentration and gradient estimation and the RLS algorithm for identifying diffusion coefficients. In the discrete simulation environment, concentration estimates are interpolated across the formation’s view-scope. Mode transitions are based on the formation’s state and the concentration estimates at its center. Figure 2 provides an overview of all major components in our algorithm and their interaction within the two operation modes. In the following sections, we will provide details of the algorithms developed for the two modes.

### 4.1 Source mapping mode

As discussed in the high-level overview, the goal of the formation in Source Mapping mode is to move toward the source of a diffusion field and facilitate diffusion field reconstruction by estimating advection and diffusion parameters. For this purpose, we train a PPO model that takes the field information vector state to predict the optimal action. In this section, we describe the setup of our training environment and the architecture of our PPO model.

We define the observation input state S(k) for our PPO model as follows:

$$S\left(k\right)=\left[z\left(r_{c}^{k},k\right),\nabla z_{x}\left(r_{c}^{k},k\right),\nabla z_{y}\left(r_{c}^{k},k\right)\right],$$ (6)

where $\nabla z_{x}\left(r_{c}^{k},k\right)$ and $\nabla z_{y}\left(r_{c}^{k},k\right)$ represent the estimated concentration gradients at the formation center at time step k , in the x and y directions, respectively. As discussed in the previous section, we rely on the CKF to provide estimates of the field concentration and gradients at the formation center, which form the complete input state of our model. By incorporating concentration gradients in the observation state, we provide the model with the direction of the largest concentration value change at the current location, which can be useful for heading toward the source center. Additionally, our definition of the state vector S(k) in Equation 6 limits the model to learning state-action values solely based on the characteristics of the field. With the formation controller, the mobile sensor robots can maintain a constant desired formation while traversing the environment; thus; we only plan the path for the formation center instead of individual robots.

For every time step, our robot formation can move to any adjacent cells (including diagonal) or stay at the current location. Thus, we can define the action space A(k) consisting of nine actions as follows:

$$A\left(k\right)=\left\{\;“\text{up}”\text{,}\;“\text{down}”\text{,}\;“\text{left}”\text{,}\;“\text{right}”\text{,}\;“\text{up}-\text{left}”\text{,}\;“\text{up}-\text{right}”\text{,}\;“\text{down}-\text{left}”\text{,}\;“\text{down}-\text{right}”\text{,}\;“\text{stay}”\;\right\}.$$ (7)

**FIGURE 3:**
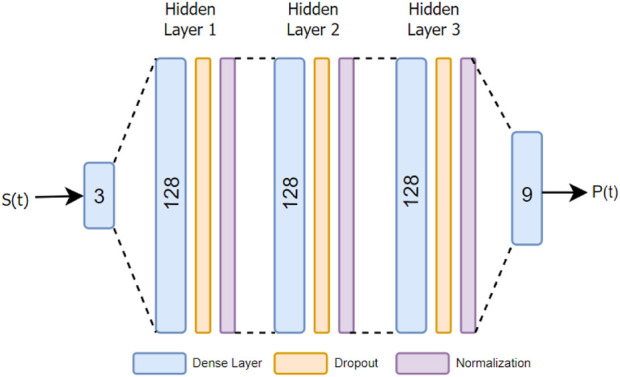
The PPO neural network architecture used in the source mapping mode.

Since our goal is to train a PPO model that can guide the formation toward the center of a diffusion field and maintain stationary state as long as possible, it is crucial to develop a reward function that incentivizes this behavior. For this reason, we model the reward function based on the concentration values inside the formation view-scope as follows:

$$R\left(k\right)=\alpha \underset{r\in \Gamma \left(k\right)}{\sum }z\left(r^{k},k\right),$$ (8)

where α is a rescale constant. As regions with high concentration values play a significant role in field reconstruction, a reward proportional to the total concentration values within the view-scope as shown in Equation 8 motivates the model to learn to navigate towards areas with higher concentration values. This, in turn, greatly reduces the error in reconstruction and prioritizes information-rich trajectory. We chose PPO as our model due to its long-standing role as a crucial component in various state-of-the-art solutions in reinforcement learning. Furthermore, PPO can be used for environments with discrete action space, as in our case. Figure 3 shows the architecture of our PPO model. Since PPO is a type of actor-critic algorithm, it has two neural networks - the actor network and critic network. In our case, we employed the same architecture for both. This network consists of three dense layers of size 128 each, an input layer of size 3 and an output layer of size 9. We added random dropout layers and regularization between the hidden layers, with ReLu as the non-linear activation function (Agarap, 2018). The network is trained using Adams optimizer (Kingma and Ba, 2015).

Using the trained PPO model, the robot formation takes actions chosen from the action space in Equation 7, and is guided toward the source of the diffusion field until it reaches the stationary state. This stationary state is achieved when the estimated field concentration reaches a local maximum and the estimated field gradient approaches zero, i.e., $z\left(r_{c}^{k},k\right)>z\left(r_{c}^{k-1},k-1\right)$ , $z\left(r_{c}^{k},k\right)>z\left(r_{c}^{k+1},k+1\right)$ , and $\nabla z\left(r_{c}^{k},k\right)\approx 0$ . At this point, the formation moves at the same speed as the advection flow for a designated period of T steps before switching to the Field Exploration mode to search for other diffusion fields in unexplored areas.

### 4.2 Field exploration mode

Whenever the robot formation reaches a stationary state, indicating the detection of the source of a diffusion field, the robot formation switches back to Field Exploration mode and moves toward unexplored area in the map to look for the remaining diffusion fields. During this phase, it is essential for the robot formation to come up with a new destination that is away from the already explored locations to avoid revisiting the same source, but also not too far to cause the formation to go back and forth when scanning the whole map. In short, our main objective is to generate a path that allows our robot formation to scan the whole field as quickly as possible without leaving any diffusion field undetected. Lawn mowing is a good example of generating a deterministic trajectory for scanning an unknown map. However, since our mobile sensing robots are often deployed in time-critical missions. It is necessary to opt for a more aggressive exploration strategy that allows the formation to discover all sources as quickly as possible. In our algorithm, we partition the unvisited cells in the entire map into multiple clusters using the K-Means clustering algorithm (Na et al., 2010). The selection of K directly affects how aggressive or cautious the scanning behavior of our robot formation will be. The initial value of K is selected based on the initial estimate of the minimum size of a diffusion field. In this work, we use the term “size” to refer to the bounded area of a diffusion field where the concentration value exceeds a certain threshold. For different scenarios (such as wildfires and gas leaks), we are often able to come up with a rough estimation of the average size of a diffusion field. Let us denote this as ${\tilde{S}}_{\mathrm{field}}$ and the global map size as $S_{\mathrm{map}}=E\times F$ . Then, K is estimated as:

$$K=\max\left(2,\left\lfloor \frac{S_{\mathrm{map}}}{{\tilde{S}}_{\mathrm{field}}}\right\rfloor \right).$$ (9)

After partitioning the map, the robot formation selects the centroid of the nearest cluster as the new destination and moves toward that destination to explore the field. It continues to visit the centroids of other clusters, prioritizing nearby clusters, as long as it is in the Field Exploration mode. The formation switches to Source Mapping mode when the estimated field concentration value exceeds a chosen threshold, i.e., $z\left(r_{c}^{k},k\right)>\delta$ . At all times, the formation maintains a record of visited clusters and diffusion fields, effectively creating a mask that distinguishes between explored and unexplored regions.

Whenever a switch occurs from Field Exploration mode to Source Mapping mode and the formation reaches the stationary state in the Source Mapping mode (indicating a new diffusion field is detected), the robot formation evaluates the field and computes a new estimated K value to repartition the remaining unexplored areas in the map. The “size” of the newly discovered diffusion field can be roughly estimated based on the circular area with radius $R_{\mathrm{dist}}$ extending from the source center to the location where the concentration first surpasses the threshold. This is also where the formation switches from Field Exploration to Source Mapping mode. Denote the size as $S_{\mathrm{new}}$ represented as:

$$S_{\mathrm{new}}\approx \pi *R_{\mathrm{dist}}^{2}.$$ (10)

With the size of the latest detected diffusion field calculated based on Equation 10, we can update the estimated average size of the diffusion field ${\tilde{S}}_{\mathrm{field}}$ as follows:

$${\tilde{S}}_{\mathrm{field}}^{\prime }=\left(1-\beta \right)\cdot {\tilde{S}}_{\mathrm{field}}+\beta \cdot S_{\mathrm{new}},$$ (11)

where β∈[0,1] is the weighting factor that determines the influence of the newly discovered field on the updated estimate ${\tilde{S}}_{\mathrm{field}}^{\prime }$ . This approach allows for increasing accurate field size estimation with new discovery. Note that K will only decrease or maintain unchanged with each new source found. When the size of an unexplored area in the map falls below a certain threshold, we consider the whole map has been adequately covered. At this point, The robot formation can either be set to idle mode or be directed to transition to a new map (potentially a neighboring global field) to initiate its operations from a different starting location. The exploration strategy can be summarized in Algorithm 2.

Algorithm 2 K-Means Clustering Based Exploration Mode.

1: **Input:** Unexplored region $M_{0}$ as an N×N grid map.
2: **Initialize:**
3: Compute initial estimate $K_{0}$ based on diffusion field radius using Equation 9.
4: Apply K-Means Clustering with $K=K_{0}$ to partition $M_{0}$ into $K_{0}$ clusters.
5: Let $A_{\text{centroids}}$ be the set of centroids for these clusters.
6: Set concentration threshold δ for mode switching.
7: Set target destination $Target=\left(x_{k},y_{k}\right)=r_{c}^{k}$ .
8: Let DONE ← False.
9: **while** $z\left(r_{c}^{k},k\right)<\delta$ **do**
10:  **if** formation center $r_{c}^{k}=Target$ **then**
11:   **if** $A_{\text{centroids}}$ ≠ ∅ **then**
12:    Set Target as the nearest centroid $C_{\text{nearest}}$ in $A_{\text{centroids}}$ .
13:    Remove $C_{\text{nearest}}$ from $A_{\text{centroids}}$ .
14:   **else**
15:     DONE ← True
16:     **break**
17:  Move formation towards Target using A* path-finding algorithm.
18: **if** DONE **then**
19:  Transition to Source Mapping Mode.
20: **else**
21:  Move to new region $M_{i}$ and restart exploration.

### 4.3 Parameter estimation and field reconstruction

**FIGURE 4:**
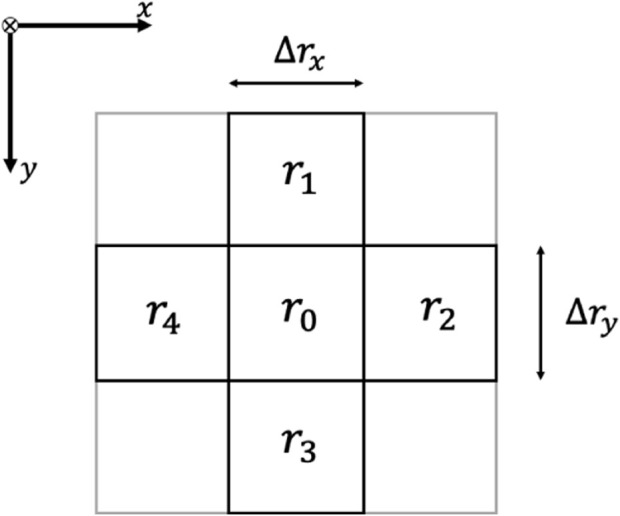
A 3×3 section of the discretized advection-diffusion field.

The field reconstruction begins when the formation detects the source of a diffusion field within the global domain. Given that our environment is modeled as a 2D grid, we discretize the diffusion Equation 1 to enable field reconstruction. Assuming the domain of interest Ω is divided into square cells of size $\Delta r_{x}=\Delta r_{y}$ , as illustrated in Figure 4, where a 3×3 grid is demonstrated. Here, $r_{1},r_{2},r_{3},r_{4}$ are the neighboring cells of grid cell $r_{0}$ . Let $z\left(r_{0},k\right),z\left(r_{1},k\right),z\left(r_{2},k\right),z\left(r_{3},k\right)$ and $z\left(r_{4},k\right)$ denote the concentration values at grid cells $r_{0}$ to $r_{4}$ at discrete time step k . Using the finite difference method, the discretized advection-diffusion equation can be expressed as:

$$\begin{array}{ll} \frac{z\left(r_{0},k+1\right)-z\left(r_{0},k\right)}{t_{s}} & =\theta \left[\frac{z\left(r_{2},k\right)+z\left(r_{4},k\right)-2z\left(r_{0},k\right)}{\Delta r_{x}^{2}}\right.\\ & \;\left.+\frac{z\left(r_{1},k\right)+z\left(r_{3},k\right)-2z\left(r_{0},k\right)}{\Delta r_{y}^{2}}\right]\\ & \;+v^{T}\nabla z\left(r_{0},k\right)+e\left(r_{0},k\right), \end{array}$$ (12)

where $t_{s}$ denotes the sampling interval and $e\left(r_{0},k\right)$ represents the approximation error. With the symmetric property, Equation 12 can be further simplified to:

$$\frac{z\left(r_{0},k+1\right)-z\left(r_{0},k\right)}{t_{s}}=\theta \frac{\sum_{i=1}^{4}z\left(r_{i},k\right)-4z\left(r_{0},k\right)}{\Delta r_{x}^{2}}+v^{T}\nabla z\left(r_{0},k\right)+e\left(r_{0},k\right).$$ (13)

To reconstruct a diffusion field using the measurements taken by the robot formation with Equation 13, we need the estimated field concentration values $\hat{z}\left(r,k\right)$ within the view-scope of the robot formation at each time step k , as well as the estimated diffusion coefficient $\hat{\theta }$ and the advection vector $\hat{v}$ . The former can be obtained through interpolation at each time step using the measurements taken by the robots. As mentioned previously, we employ the strategy developed in (Wu et al., 2020) to identify the diffusion coefficient θ . In many scenarios, the advection coefficient v is assumed to be a known constant. When the advection coefficient is unknown, we estimate it when the robot formation reaches a stationary state, where the robot’s velocity matches that of the advection flow. Let $v={\left(v_{x},v_{y}\right)}^{T}$ and $k_{S}>0$ represent the time step when a stationary state is detected. Assuming the formation stays at the stationary state for T steps, $\left(v_{x},v_{y}\right)$ can be estimated as

$$\begin{array}{cc} & {\hat{v}}_{x}=\frac{r_{c,x}^{k_{S}+T}-r_{c,x}^{k_{S}}}{T},\\ & {\hat{v}}_{y}=\frac{r_{c,y}^{k_{S}+T}-r_{c,y}^{k_{S}}}{T}. \end{array}$$ (14)

With these values determined, the field values across the diffusion field can be propagated through Equation 13. This approach enables field reconstruction using only the sparse measurements gathered along the robots’ paths.

## 5 Simulation results

In this section, we provide a comprehensive analysis of the proposed multi-robot field reconstruction strategy, which encompasses source mapping and field exploration modes in simulations. We begin by outlining the implementation details, followed by a discussion on the PPO training specifics. Finally, we present the results derived from these simulations.

### 5.1 Simulation environment

**FIGURE 5:**
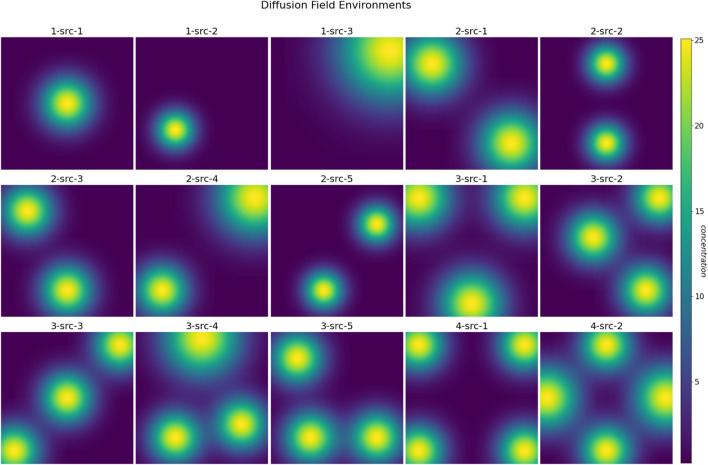
A set of 15 simulation environments, each consisting of one to four diffusion fields centered at various locations with distinct diffusion and advection coefficients.

**FIGURE 6:**
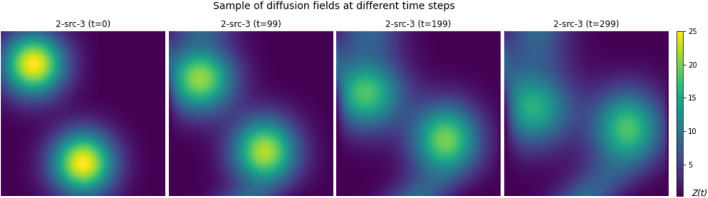
Sample of a diffusion field environment over different time steps.

To assess the overall solution, we developed a low-fidelity simulation environment within a discrete space. This environment is structured as a 100×100 square grid, incorporating between one to four non-overlapping spatial diffusion fields of varying sizes and configurations, as depicted in Figure 5. We generated a total of 15 different environments, with increasing level of complexity, to investigate and assess the model’s efficiency in mapping unknown environments. The color of each cell in the grid denotes the concentration value of the diffusion field. Each diffusion field possesses distinct and independent advection and diffusion coefficients. Figure 6 shows the evolution of a sample diffusion field over time. The environment was simulated for up to 400 time steps with the discrete interval Δt=0.1 , Δx=0.1 , and Δy=0.1 . Table 1 lists the configurations of the 15 diffusion field environments with advection terms $\left(v_{x},v_{y}\right)$ and diffusion term θ . The center of the field is denoted as pos(x,y) . Note that size is only used internally by the generator as a scale factor to control how large a diffusion field appears on the map.

**TABLE 1:** Configurations of the 15 diffusion field environments with advection terms $\left(v_{x},v_{y}\right)$ and diffusion term θ . The center of the field is denoted as pos(x,y) . Note that size is only used internally by the generator as a scale factor to control how large a diffusion field appears on the map.

Source	pos (x,y)	Size	$v_{x}$	$v_{y}$	θ
1-src-1	(50, 50)	80	0.73	−0.44	0.76
1-src-2	(30, 70)	60	0.14	−0.65	1.02
1-src-3	(90, 10)	160	0.74	0.09	1.24
2-src-1	(20, 20)	100	0.62	−0.01	1.07
	(80, 80)	100	−0.04	−0.14	0.96
2-src-2	(50, 20)	60	−0.22	−0.47	1.04
	(50, 80)	60	−0.59	−0.45	1.08
2-src-3	(20, 20)	80	−0.71	0.09	1.17
	(50, 80)	80	0.56	−0.67	0.78
2-src-4	(20, 80)	80	−0.20	0.37	0.75
	(90, 10)	120	0.71	0.78	0.99
2-src-5	(40, 80)	60	0.10	0.38	1.15
	(80, 30)	60	−0.09	0.25	1.07
3-src-1	(10, 10)	100	0.54	0.19	1.21
	(50, 90)	100	0.03	0.58	0.94
	(90, 10)	100	−0.64	0.67	0.79
3-src-2	(40, 40)	80	0.39	−0.50	0.81
	(80, 80)	80	0.60	0.25	0.93
	(90, 10)	80	−0.12	0.11	1.18
3-src-3	(50, 50)	80	−0.37	0.24	1.13
	(10, 90)	80	−0.32	−0.75	1.21
	(90, 10)	80	0.76	0.71	1.02
3-src-4	(50, 5)	120	−0.49	0.17	0.91
	(30, 80)	80	−0.50	0.56	0.84
	(80, 70)	80	−0.44	0.42	0.98
3-src-5	(20, 20)	80	0.58	0.45	0.97
	(80, 80)	80	0.66	−0.18	0.99
	(30, 80)	80	0.73	−0.23	0.99
4-src-1	(10, 10)	80	−0.08	0.22	1.14
	(90, 10)	80	0.37	0.35	0.77
	(90, 90)	80	−0.72	−0.38	0.76
	(10, 90)	80	0.37	0.63	0.86
4-src-2	(50, 10)	80	0.72	−0.73	1.05
	(50, 90)	80	−0.75	0.74	1.06
	(5, 50)	100	0.35	−0.05	1.03
	(95, 50)	100	0.12	0.73	1.25

Since the map is discretized in our approach, selecting an appropriate grid cell size also plays a role in both the accuracy of data collection and computational efficiency. Each grid cell should be small enough to capture meaningful concentration gradients but large enough to reduce computational demands. Ideally, the grid cell size should reflect both the overall map dimensions and the characteristics of the environment being monitored. For example, when studying gas leaks, where subtle concentration changes are significant, a finer grid may be required. On the other hand, wildfire propagation fields, which tend to cover larger areas, can accommodate slightly larger cells. Adjusting the grid cell size based on the specific characteristics of the environment allows us to find the right balance between resolution and computational cost, facilitating effective and efficient exploration and reconstruction.

### 5.2 PPO training

For the training of the PPO model, we follow a curriculum learning approach (Wang et al., 2023; Wang et al., 2022) that involves gradually increasing the complexity of the training environment. We created a 100×100 training environment featuring a single diffusion field at the center of the map p=(50,50) with a constant diffusion coefficient θ=1 . The spread of this diffusion field at the beginning of an episode is drawn randomly based on the parameter size , which controls how large the area with non-zero concentration is due to the presence of the diffusion field. This environment has two variants: a “static” environment with the advection term set to zero, and a “dynamic” environment with a fixed non-zero advection term.

We simulate a group of four mobile robots in a symmetric formation as shown in Figure 1 to move in the environments, with the formation controller running to maintain the desired formation. With the CKF providing the state S(k) which includes the estimated field value $z\left(r_{c}^{k},k\right)$ and gradient along the trajectory of the formation center $\nabla z_{x}\left(r_{c}^{k},k\right)$ and $\nabla z_{y}\left(r_{c}^{k},k\right)$ , the PPO model underwent training on static maps first before advancing to training on dynamic maps. After training, our PPO model outputs the policy to direct the robot formation to move toward the source of a diffusion field and maintain the stationary state, which is required for the estimation of advection coefficients.

**FIGURE 7:**
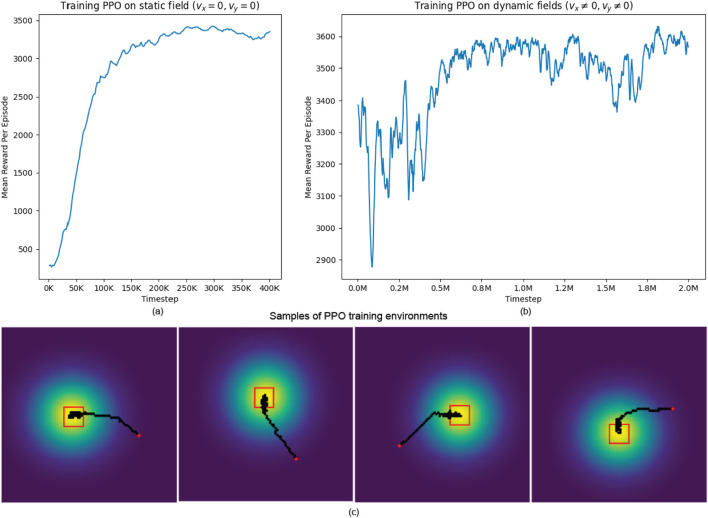
**(A)** Stage 1: Pre-training PPO on static fields. **(B)** Stage 2: Training PPO on dynamic fields. **(C)** Samples of training environments and generated trajectories post-training.

We provide the training results in Figure 7. The PPO model was initially trained for 400,000 time steps on the static environment where advection terms are set to zero, as shown in Figure 7A. After that, we proceeded to train the PPO model on the dynamic environment for an additional 2,000,000 time steps, as shown in Figure 7B The model was trained multiple times with orthogonal random weight initialization using the Stable-Baselines3 framework (Raffin et al., 2021), and the best-performing model was selected for use in our experiments. In both environments, the formation is initially placed in a low-concentration region at the start of each episode to avoid starting too close to the source. Additionally, in our dynamic environment, the source is assigned random, non-zero advection and diffusion coefficients, causing it to move in a different direction in each episode. This setup encourages the model to adapt to various scenarios but also introduces some fluctuations in performance early in training, as the formation may take suboptimal actions initially and struggle to catch up to the moving source. In both training phases, however, the average reward per episode increases steadily, indicating that the model successfully improves its given task over time. Additionally, Figure 7C provide some samples of source-heading operation performed by our PPO model post-training. In these samples, the 4-robot formation, shown as the red square in the map, are tasked with locating the source while maintaining its formation. The yellow region denotes the area with high concentration - where the source is located, the red square represents the robot formation, and the red dot is the starting position of the formation center. The robot formation is directed towards the center of the source, which has maximum concentration, per the PPO’s objective of reward maximization. PPO’s role in this task is to update the policy per iteration to make an informed decision on where to go next. The simulation results show the PPO algorithm’s efficacy in source mapping.

The PPO algorithm’s training process effectively learns a policy that guides the robot formation to explore the field, detect diffusion sources, and assist in reconstruction while adapting to dynamic changes in the environment. The algorithm’s capability in handling complex and dynamic environments indicates its potential in real-life scenarios where rapid environmental change happens, and accurate detection of diffusion sources is crucial. This capability is precious in pollution tracking or gas leak detection scenarios, where time-sensitive and precise localization is essential.

### 5.3 K-means clustering-based exploration

When the PPO model leads the robot formation to move toward a diffusion source and the formation center reaches the stationary state, the advection coefficient can be estimated based on Equation 14, and the field reconstruction process can start using Equation 13. Along with the field reconstruction process, the robot formation switches to the field exploration mode, where the K-means clustering-based exploration algorithm 2 plays a role.

**FIGURE 8:**
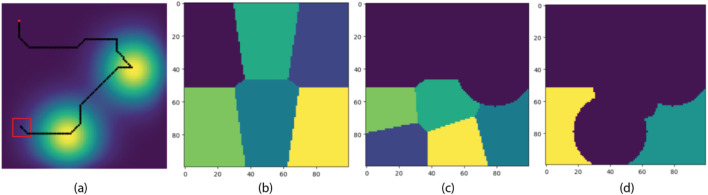
Field Re-partitioning Behavior of the Exploration Module using K-Mean Clustering. The red dot is the starting position of the formation center. Partitioning happens at the beginning of an episode and whenever a new source is detected. **(A)** The trajectory of the formation center. **(B)** The initial partition of the field. **(C)** The updated partition after the first source on the upper right corner is detected. **(D)** The updated partition after the second source on the lower left corner is detected.

Figure 8 provides an example of how the K-means clustering-based exploration works in a 100×100 grid map with two diffusion fields. The formation started in Field Exploration mode with the starting position shown as the red dot. Based on our initial assumption about the average size of diffusion fields, the map is partitioned into six clusters with K=6 as illustrated in Figure 8B. The formation moves toward the centroid of the nearest cluster and continues to other neighboring centroids until an area with a high concentration is detected. When that happens, the formation transitions to Source Mapping mode and attempts to move toward the source center to reconstruct the field. When the new diffusion field is fully reconstructed, the formation switches back to Field Exploration mode, re-calculates a new K as described in Equations 9-11 and re-partitions the unexplored area, as shown in Figure 8C. Note that the unexplored area has excluded visited clusters and any detected diffusion fields. This process continues to repeat until the map is fully covered.

### 5.4 Multi-robot source seeking and field exploration results

**FIGURE 9:**
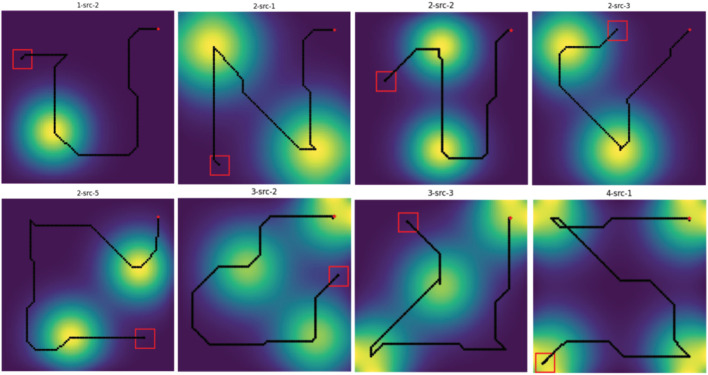
Generated trajectories obtained from simulations across different spatial-diffusion environments. The robot formation center is initially located at $r_{c}=\left(90,10\right)$ - shown as the red marker, and the red square shows the position of the formation the end of the episode.

With the trained PPO model and the K-means clustering-based exploration algorithm, we now ready to implement the overall multi-robot Source Mapping and Field Exploration strategy to reconstruct a dynamic field. Figure 9 shows different trajectories of the robot formation obtained from the simulation across multiple spatial-diffusion environments. In this setup, the robot formation started at the same initial position $r_{c}^{0}=\left(90,10\right)$ (top-right corner, shown as a red marker). The red square displays the final position of the robot formation at the end of the episode (k=300) . Despite variations in spatial-diffusion field distributions and characteristics, we can see that the robot formation managed to detect all sources in the map while efficiently covering the entire map before the episode ends.

**FIGURE 10:**
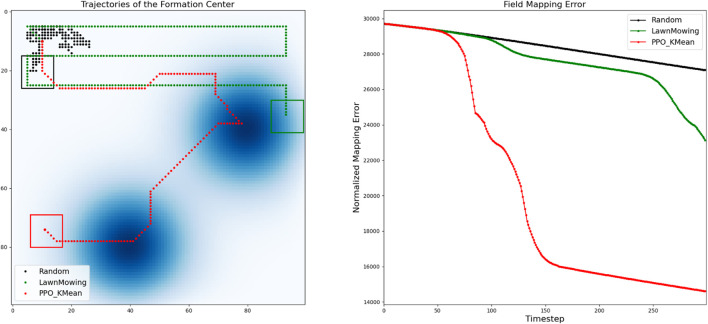
Analysis of the generated trajectory and mapping errors of our solution in comparison with the Lawn Mowing and Random Walking approaches.

In Figure 10, we compare the trajectory and mapping errors of our solution against other alternative path-planning algorithms, including Random-Walking (shown in black) and Lawn-Mowing (shown in green). With the Random-Walking strategy, the formation takes a random action in every time-step, resulting in an arbitrary trajectory. On the other hand, with Lawn-Mowing strategy, the formation attempts to scan the field row-by-row until the entire field is fully covered. Compared to these approaches, the trajectory generated by our solution is faster at detecting and mapping diffusion fields, resulting in a much lower mapping error. In this map, our K-Mean-based approach is the only one that detects all diffusion fields before the episode ends (k=300) .

## 6 Experimental results

**FIGURE 11:**
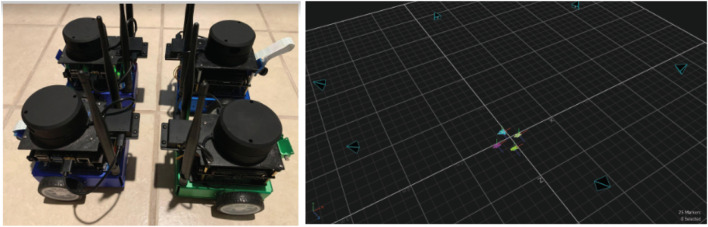
Mobile robot formation setup for real-world testing and evaluation.

To validate our solution in a real-world setting, we developed a high-fidelity testing environment in our lab. Our setup includes four mobile robots operating in a 12 × 12 square foot open field that simulates an actual advection-diffusion environment. Figure 11 shows our laboratory setup of the mobile sensor network consisting of 4 mobile robots with motion tracking enabled, allowing for accurate collection of real-time trajectory data. The robots are two-wheel differential drive and ROS-based, running on the Jetson Nano (Developer Nvidia, 2024) computing platform. Each robot is equipped with a 2D Lidar scanner [YDLidar-G4 (YDLIDAR-G4-Datasheet, 2024)], a speed encoder, and an IMU (BNO-055 (Industries, 2024)). To enable low-latency sensor fusion, a Teensy 4.0 (PJRC, 2024) collects and preprocesses sensor readings from the speed encoder and IMU before streaming the results to the main board via rosserial. Lidar is installed to enable basic obstacle avoidance behaviors, allowing the formation to adapt to various scenarios when navigating in outdoor environments.

For localization, we rely on an indoor motion capture system to provide absolute positional tracking, analogous to GPS in outdoor scenarios. An Extended Kalman Filter (Ribeiro, 2004) fuses data from both the IMU and motion capture system to improve real-time position estimation of the robots. Our software stack uses ROS Noetic (Quigley et al., 2009) and its ecosystem to facilitate sensor fusion for localization and obstacle avoidance, as well as to simulate and visualize the behaviors of the advection-diffusion field. In our stack, each robot has its own action server (based on ROS Action), which is responsible for moving the robot to a target destination. A master node running on a stand-alone computer is responsible for broadcasting the concentration values of the simulated field as well as performing formation control during the experiment.

**FIGURE 12:**
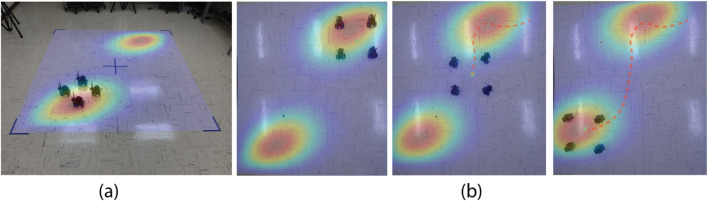
**(a)** Lab experiment setup with a projected dynamic field and four mobile robots. **(b)** Snapshots of the trajectories of the robot formation at three different time steps in an experiment. The red dashed lines represent the trajectories.

Figure 12 presents various views of the simulated spatial-temporal diffusion field in our laboratory as well as an example of the trajectory generated by our robot formation. Given the difficulties of installing physical diffusion field sources indoors, we utilized computational models to simulate the environment. The simulated field is projected onto the floor in real-time footage captured by side and top-down cameras. Sensor measurements are generated based on the robots’ locations, which are tracked using the motion capture system.

**FIGURE 13:**
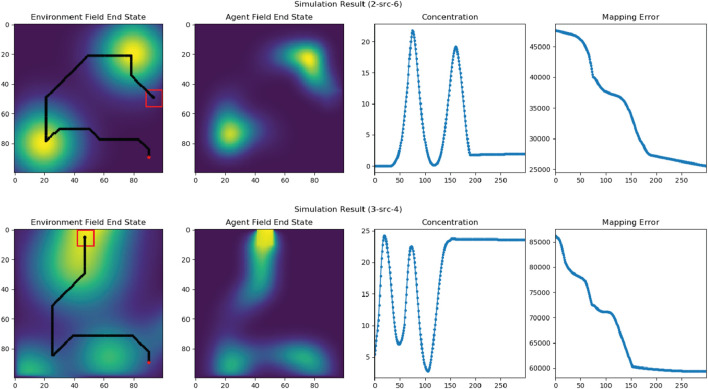
The field exploration and reconstruction results in two experiments with two and three diffusion sources. “Environment Field End State” figures illustrate the end states of the two experiments with corresponding trajectories of the robot formation. The red dots indicate the starting locations of the formation center and the red squares are the ending locations of the formation. “Agent Field End State” figures show the end states of the reconstructed fields in the two experiments. “Concentration” figures illustrate the estimated field concentration along the trajectories of the formation center, and “Mapping Error” figures show the mapping errors while reconstructing the fields.

**FIGURE 14:**
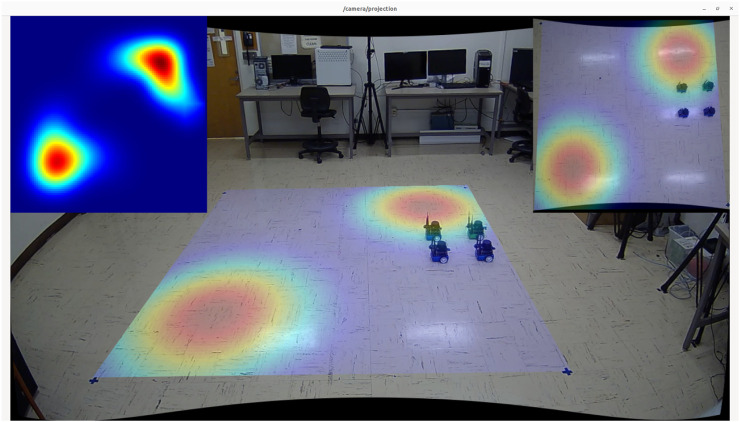
Screenshot captured from the experiment on the first environment with two diffusion fields.

**FIGURE 15:**
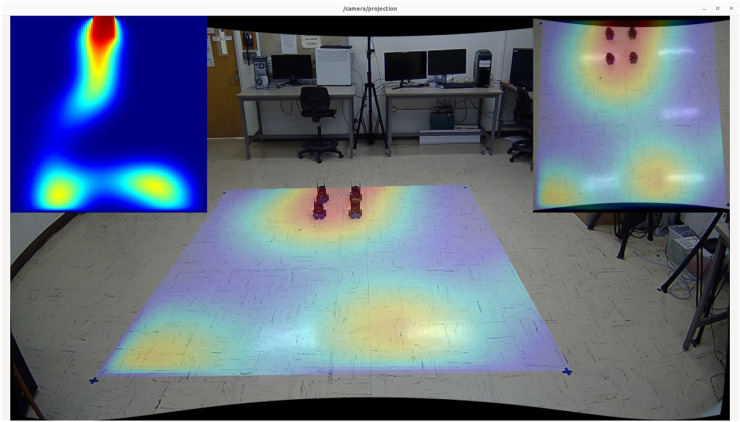
Screenshot captured from the experiment on the second environment with three diffusion fields.

To validate our solution in real-world settings, we selected two spatial-diffusion environments from our list and ran simulation experiments using actual mobile robots. While the spatial-temporal diffusion field is generated by computer simulation, the mobile sensor network is still designed to function exactly like how they should behave in the real-world. This involves having individual mobile robots take raw measurements and combine the results to estimate the concentration and gradients at the formation center, using CKF. Figure 13 provides a summary of our experimental results. The first column “Environment Field End State” shows the final states of our spatial diffusion environments and the trajectories of the robot formation. In both experiments, the formation enters the map from the bottom right corner with $r_{c}^{0}=\left(90,90\right)$ - shown as a red marker, and the red square again shows the formation’s final location when the episode ends. The “Agent Field End State” column shows the final reconstructed field computed by our mobile sensor network. As we can see, the formation center managed to explore the entire map while following an information-rich trajectory, which resulted in consistently high concentration readings and low mapping errors. Additionally, the results from experiments in the high-fidelity simulation environment closely resemble those from the low-fidelity testing environment, demonstrating that our solution can be adapted to more realistic scenarios. Additional screenshots from our laboratory experiments are available in Figures 14, 15. The plots in the upper left corners in both figures illustrate the reconstructed fields.

## 7 Conclusions and future work

In this paper, we developed a strategy to map and reconstruct dynamic fields with multiple diffusion sources using a multi-robot formation. This strategy proved effective on various maps with different configurations. Our approach efficiently explores unknown maps while ensuring that potential diffusion sources are detected. The results from our experiments show that the robot formation can effectively utilize environment data from all robots to navigate toward the source center and accurately reconstruct the advection and diffusion coefficients. While we did encounter some challenges with overlapping diffusion fields, these complexities only underscore the need for further research and detailed experiments. Our system holds potential for practical use in scenarios like rescue missions and field explorations, where robots can assess hazards before sending humans into these environments. This research shows the capability and versatility of our multi-robot system in environmental monitoring and could be important in enhancing safety measures during high-risk missions.

## Data Availability

The raw data supporting the conclusions of this article will be made available by the authors, without undue reservation.
